# Free 25(OH)D3 levels in follicular ovarian fluid top-quality embryos are higher than non-top-quality embryos in the normoresponders group

**DOI:** 10.1038/s41598-024-71769-6

**Published:** 2024-11-22

**Authors:** Artha Falentin Putri Susilo, Hanom Husni Syam, Hartanto Bayuaji, Anita Rachmawati, Binarwan Halim, Wiryawan Permadi, Tono Djuwantono

**Affiliations:** 1 Department of Obstetrics and Gynaecology, Faculty of Medicine Universitas Padjadjaran - Dr. Hasan Sadikin General Hospital, Pasteur No. 38, Bandung, West Java 40161 Indonesia; 2 Department of Obstetrics and Gynaecology, Faculty of Medicine Universitas Sumatera Utara – H Adam Malik Hospital, Medan, Indonesia; 3 Bandung Fertility Center, Limijati Hospital, Bandung, Indonesia; 4 Department of Obstetrics and Gynaecology, Universitas Prima Indonesia, Medan, Indonesia

**Keywords:** Vitamin D, Embryo, In vitro fertilization, Top-quality embryo, Cell biology, Biomarkers

## Abstract

Vitamin D and calcium in follicular fluid play an important role in modulating steroidogenesis, folliculogenesis, and oocyte quality determination. Both collaborate to produce top-quality embryos (TQE) during in vitro fertilization (IVF). In this study, we compared free 25(OH)D3 and calcium levels in follicular fluid between TQE and non-TQE groups. This cross-sectional study included women who underwent IVF procedures at tertiary hospitals in Bandung, Indonesia. Ovarian follicular fluid was collected during the ovum pick-up procedure. The examination of 25(OH)D3 levels, vitamin d-binding protein, and calcium in the follicles was done using an enzyme-linked immunosorbent assay (ELISA). Free 25(OH)D3 levels were calculated using the Vermeulen formula. A total of 173 samples met the study criteria, including 86 subjects in the TQE group and 87 subjects in the non-TQE group. There was a significant difference in free 25(OH)D3 follicular fluid levels between the TQE and non-TQE groups (p = 0.017); however, there was no significant difference in calcium levels between the two groups (p = 0.805). We also found that there was a significant association between free 25(OH)D3 follicular fluid levels and embryo quality (OR 3.05, 95% CI 1.46–6.38; p-value = 0.002); however, there was no significant association between follicular fluid calcium and embryo quality [p = 0.144 and OR, 1.74 (95% CI 0.82–3.68)]. The results suggest that free 25(OH)D3 and calcium in the follicular fluid act independently during steroidogenesis, folliculogenesis, and fertilization.

## Introduction

Vitamin D is a steroid hormone that plays an essential role in various physiological processes, particularly calcium and phosphorus homeostasis and bone mineralization^1–7^. Vitamin D also plays a pivotal role in human reproductive functions, such as folliculogenesis, steroidogenesis, and successful embryo implantation^3,4^. Thus, vitamin D levels may be a predictor of the success of assisted reproductive technology^4,6^.

The effects of vitamin D and calcium on oocyte development have important clinical implications for the success of in vitro fertilization (IVF). Vitamin D deficiency can lead to calcium deficiency, which results in disturbances in oocyte maturation, development, and fertilization^8–10^.

Vitamin D plays a role in follicle regulation and oocyte maturation through direct and indirect pathways^4^ and contributes to embryogenesis and folliculogenesis. Vitamin D modulates primary follicle recruitment by regulating anti-Müllerian hormone (AMH) production^11^.

Thus, intrafollicular vitamin D levels may be a potential marker of oocyte and embryo quality as well as a predictor of IVF outcomes^4^. Moreover, free vitamin D3 and bioavailable vitamin D3 levels may be a more accurate predictor in assessing vitamin D status^12^. Free 25(OH)D3 and calcium levels in follicular fluid may play a role in the final maturation of follicles and are associated with the rate of fertilization, cleavage, and the quality of the embryo, thus increasing the rate of pregnancy; however, their exact roles in stimulating oocyte and embryo quality have not been adequately reported.

In this study, we compared free 25(OH)D3 and calcium levels in follicular fluid between top-quality embryo (TQE) and non-top-quality embryo (non-TQE) groups. We also determined the correlation between free 25(OH)D3 and calcium levels in follicular fluid and embryo quality in normoresponder patients who underwent IVF procedures.

## Material and methods

### Study design

This observational cross-sectional study used secondary data from the ovarian follicular fluid of women who underwent IVF in tertiary hospitals in Bandung, Indonesia. This study was conducted according to the Declaration of Helsinki of the World Medical Association. All methods were performed according to relevant guidelines and regulations after obtaining approval and recommendations from the Ethics Committee Review Board of Hasan Sadikin General Hospital, Faculty of Medicine Universitas Padjadjaran (reference number LB.02.01/X.6.5/399/2022).

### Research subjects

The subjects included women who underwent IVF procedures at the Aster Clinic, Dr. Hasan Sadikin General Hospital Bandung, and Bandung Fertility Center Limijati Hospital Bandung between November 2021 and March 2022 and who met the inclusion and exclusion criteria. The inclusion criteria were as follows: women of reproductive age (20–35 years old) who belonged to the normoresponder group, a body mass index (BMI) varying between 18 and 25 kg/m^2^, participated in an IVF program for the first time, no exposure to toxic substances (cigarettes, alcohol), and no infection or previous ovarian surgery. Subjects in the hyper- and poor-responder groups had no oocytes in the ovarian follicular fluid. Individuals with two oocytes detected in one sample tube, severe endometriosis, adenomyosis, polycystic ovarian syndrome, fulfilled the Rotterdam criteria, or with severe sperm abnormalities were excluded.

### Sampling sizes and sampling procedures

Consecutive sampling was done from November 2021 to March 2022. Samples were collected from participants who met the study criteria. The determination of sample size was based on statistical calculations by setting a 95% confidence level and an 80% power test. With a 95% confidence level and 80% power, this study required a minimum of 86 samples per group, which equaled 172 samples in total.

### Data collecting and laboratory examination

The subjects followed an IVF program using a controlled ovarian stimulation protocol with either a short or a long protocol. A transvaginal ultrasound examination was performed on each subject before beginning the program. The short protocol began when the patient was on the 2nd day of menstruation by administering recombinant FSH at a dose of 75–450 IU/day. On the 6th or 7th day of stimulation, or when follicles ≥ 14 mm were detected, a GnRH antagonist (Cetrorelix 0.25 mg) was administered. Recombinant human chorionic gonadotropin (hCG) or a GnRH agonist was administered when at least three follicles with a diameter of 18 mm were found. Oocyte picking was done at 34–36 hours after stimulation.

The long protocol began on the 21st day of menstruation with the administration of a 0.5 mg GnRH analog until the patient was menstruating. When the patient menstruated on the first day, endometrial thickness was assessed and antral follicle counts were conducted. If endometrial thickness was less than 5 mm, stimulation was performed using recombinant FSH at a dose of 75–450 IU/day along with 0.2 mg GnRH analog. Stimulation with recombinant human chorionic gonadotropin was done when at least three follicles with a diameter of 18 mm were detected. The ovum pick-up procedure was performed 34–36 hours after treatment.

Before the ovum pick-up procedure, the size of the follicles was examined by oocyte aspiration. Follicle size was measured using two diameter measurements, which were averaged. Oocytes were collected using a 17-gauge oocyte aspiration needle under transvaginal ultrasound guidance. The follicular fluid and oocytes were aspirated without flushing and stored in a Falcon tube. Only one Falcon tube was used for each follicle. The follicular fluid was examined by an embryologist using a light microscope to identify oocytes and assess oocyte maturity. The follicular fluid in the tube containing oocytes was used as a research sample, and codes were assigned to those tubes by the examiner. The tube containing the follicular fluid was stored at – 85 °C until the levels of 25-OH-vitamin D3, vitamin D-binding protein, albumin, and calcium were measured. Each patient had a maximum of eight follicular fluid samples.

The examination of 25-OH-vitamin D3, vitamin D-binding protein, albumin, and calcium levels was performed at the Clinical Pathology Laboratory of Dr. Hasan Sadikin General Hospital, Bandung. The tube containing follicular fluid was centrifuged at 300 *rpm*, then separated into 2 aliquots containing 0.5–1 mL each. The measurement of 25(OH)D3, vitamin d-binding protein, albumin, and calcium levels in the follicles was done by ELISA. Free 25(OH)D3 levels were calculated using a method Vermeulen formula^13^ as follows:

$$ {\text{Levels of Free 25}}\left( {{\text{OH}}} \right){\text{D}}_{{\text{3}}}  = \frac{{{\text{Total 25}}\left( {{\text{OH}}} \right){\text{D}}_{{\text{3}}} }}{{{\text{1 }} + {\text{ }}\left[ {(6 \times 105 \times } \right[{\text{Albumin}}\left] {){\text{ }} + {\text{ }}(7 \times 108 \times } \right[{\text{VDBP}}\left] ) \right]}}, $$

$$ \% {\text{ Free 25}}\left( {{\text{OH}}} \right){\text{D}}_{{3}} = \frac{{{\text{Free 25}}\left( {{\text{OH}}} \right){\text{D}}_{{3}} }}{{{\text{Total 25}}\left( {{\text{OH}}} \right){\text{D}}_{{3}} }}. $$

### Study outcomes

The Kolmogorov–Smirnov test was used to assess for normality (for n > 50) and the data was considered normally distributed if p > 0.05. The Chi-Square test was used to examine the relationship between two categorical variables. To compare two means, an independent *t*-test was done or the Mann–Whitney *U* test was performed if the data did not follow a normal distribution. Cutoff values were determined using the receiver operating characteristics (ROC) curve. The Chi-Square test was used to examine the relationship between the cutoff values for free 25(OH)D3 levels and relative risk, and the 95% confidence interval (CI) was calculated to assess the effect size. Data processing and analysis were conducted using SPSS version 26.0 for Windows. The outcomes for this study were as follows: (1) differences in 25(OH)D3 and calcium levels in ovarian follicular fluid between TQE and non-TQE normoresponder women who underwent IVF procedures; and (2) correlation between free 25(OH)D3 and calcium levels in ovarian follicular fluid and embryo quality in normoresponder patients undergoing IVF.

## Results

During the study period, 173 follicular fluid samples were obtained, with 86 and 87 samples from the TQE and non-TQE groups, respectively. Table 1 lists the characteristics of the subjects. The two groups (TQE and non-TQE) were comparable in terms of age, BMI, infertility duration, total gonadotropin dose, estradiol level, progesterone level, basal AMH level, AFC, sperm analysis, and sperm count. The mean age of the patients was 30 ± 2.9 in the TQE group and 30.6 ± 2.9 in the non-TQE group (p = 0.678). The mean value of estradiol levels on the stimulation day was 4645 ± 386.7 in the TQE group and 4727.4 ± 2386.9 in the non-TQE group (p = 0.771), which indicated no statistical difference (Table 1).

**Table 1:** Comparison of patient baseline demographics and clinical parameters between the TQE and non-TQE groups.

Characteristics	Groups		p-value*
	Top-quality embryo (n = 19)	Non-top-quality embryo (n = 23)	
Age (years)			0.678
25–29	9	10	
30–35	10	13	
Mean (SD)	30 (2.9)	30.6 (2.9)	
Body mass index (kg/m^2^)			1.000
Mean (SD)	21.9 (1.1)	22.0 (1.1)	
Infertility duration (years)			0.590
Mean (SD)	5.24 (2.82)	5.72 (2.86)	
Median (range)	5 (2–12)	5 (2–12)	
Gonadotropin total dose (IU)			0.256
Mean (SD)	2877.6 (386.7)	2752.2 (466.4)	
Median (range)	2700 (2475–3600)	2700 (1650–3600)	
Estradiol level (pg/mL)			0.771
Mean (SD)	4645.0 (386.7)	4727.4 (2386.9)	
Median (range)	4096 (2703–9899)	3907.1 (2245–12,012)	
Progesterone level (ng/mL)			0.868
Mean (SD)	1.12 (0.40)	1.09 (0.41)	
AMH basal level (ng/mL)			0.830
Mean (SD)	2.36 (1.01)	2.44 (1.01)	
Median (range)	2.0 (1.23–4.16)	2.13 (1.21–4.16)	
AFC			0.691
Mean (SD)	13.37 (2.24)	13.09 (2.29)	
Type of infertility			0.936
Primary	13	16	
Secondary	6	7	
Sperm analysis			0.661
Normozoospermia	7	7	
Teratozoospermia	12	16	
Total sperm count (million)			0.695
Mean (SD)	256.2(156.6)	235.9 (153.1)	
Median (range)	192.6 (64–672)	192.6 (64–672)	

*A Chi-square test was performed on age, type of infertility, and sperm analysis variables. The duration of infertility, total gonadotropin dose, and sperm count were analyzed with the Mann–Whitney test. The other factors were analyzed by a *t*-test.

A comparison of the various study variables in the two groups is shown in Table 2. The two groups differed significantly in total follicular vitamin D levels (p = 0.009), free follicular vitamin D levels (p = 0.017), and follicle size (p < 0.001). The levels of 25-OH-vitamin D3 total follicles, 25-OH-vitamin D3 free follicles, and follicular calcium, which may be used as predictors of TQE, were analyzed using a ROC curve to determine the cutoff point, the area under the curve (AUC), sensitivity, and specificity (Table 3).

**Table 2:** Comparison of the variables examined in the TQE and non-TQE groups.

Variables	Groups		p-value*
	Top-quality embryo (n = 86)	Non-top-quality embryo (n = 87)	
Total follicular 25(OH)D_3_ (ng/mL)			
Median (range)	14.15 (0.6–98.9)	11.2 (1.8–38.0)	0.009
Follicular calcium (mg/dL)			
Median (range)	6.30 (3.74–18.49)	6.23 (3.60–17.82)	0.805
Follicular albumin (g/dL)			
Median (range)	14.0 (8.42–46.12)	14.0 (8.77–38.71)	0.998
Free follicular 25(OH)D_3_ (ng/mL)			
Median (range)	0.0012 (0.001–0.013)	0.0011 (0.0001–0.003)	0.017
% Free follicular 25(OH)D_3_			
Mean (SD)	0.00942 (0.002)	0.00918 (0.002)	0.510

*Mann–Whitney test, except for % free 25(OH)D with *t*-test.

**Table 3:** The cutoff value used as a predictor of top-quality embryos.

Variable	Cutoff value	AUC (CI 95%)	p-value	Notes
Follicular total 25(OH)D_3_	> 12.1 ng/mL	0.614 (0.538–0.687)	0.0075	Sensitivity = 59.3%
				Specificity = 62.07%
Free follicular 25(OH)D_3_	> 0.00175 ng/mL	0.605 (0.528–0.678)	0.0146	Sensitivity = 34.88%
				Specificity = 85.06%
Follicular calcium	> 4.88 mg/dL	0.511 (0.434–0.588)	0.806	Sensitivity = 83.72%
				Specificity = 25.29%

The relationship between the cutoff value for total 25-OH-vitamin D3 levels and follicle-free 25-OH-vitamin D3 levels with embryo quality was determined for the TQE and non-TQE groups in normoresponder patients undergoing IVF (Table 4). Patients with total follicular 25(OH)D3 levels of > 12.1 ng/mL had a 2.38-fold higher tendency to develop TQE. Patients with follicular free 25(OH)D3 levels > 0.00175 ng/mL had a 3.05-fold higher chance of developing TQE compared with those with 25(OH)D3 levels ≤ 0.00175 ng/mL. The cutoff value for follicular calcium levels was > 4.88 mg/dL (p = 0.144). There was no statistically significant correlation between the follicular calcium levels in the two study groups.

**Table 4:** Correlation between total 25(OH)D_3_ and free 25(OH)D_3_ levels in follicular fluid with embryo quality in normoresponder patients who underwent IVF.

Variable	Groups		p-value	OR (CI 95%)
	TQE (n = 86)	Non-TQE (n = 87)		
Total follicular 25(OH)D_3_				
> 12.1 ng/mL	51 (59.3%)	33 (37.9%)	0.005	2.38 (1.29–4.39)
≤ 12.1 ng/mL	35 (40.7%)	54 (62.1%)		
Free follicular 25(OH)D_3_				
> 0.00175 ng/mL	30 (34.9%)	13 (14.9%)	0.002	3.05 (1.46–6.38)
≤ 0.00175 ng/mL	56 (65.1%)	74 (85.1%)		
Calcium levels				
> 4.88 mg/dL	72 (83.7%)	65 (74.7%)	0.144	1.74 (0.82–3.68)
≤ 4.88 mg/dL	14 (16.3%)	22 (25.3%)		

OR (CI 95%) = odds ratio with confidence interval of 95%.

**Fig. 1:**
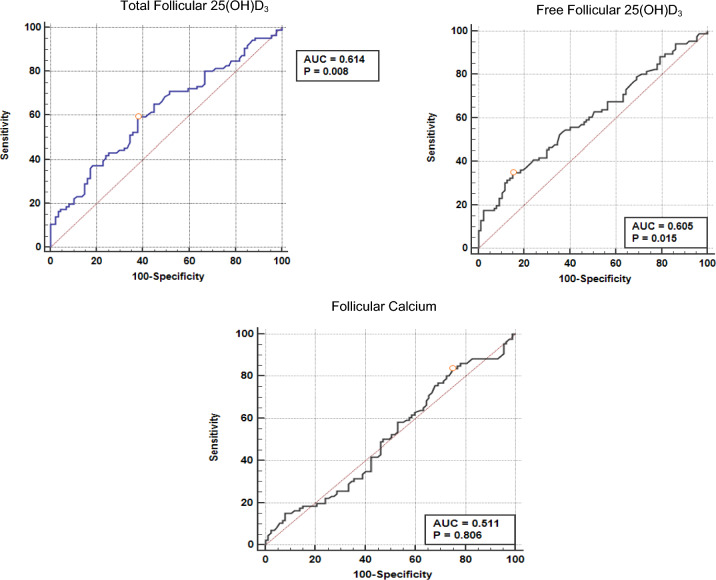
ROC curves for total follicular 25(OH)D_3_, follicular free 25(OH)D_3_, and follicle calcium.

The follicle-free 25–25-OH-vitamin D3 cutoff value > 0.00175 ng/mL can be used as a predictor of TQE. The follicle calcium cutoff value that can be used as a predictor of TQE was > 4.88 mg/dL. A follicular total 25(OH)D3 level > 12.1 ng/mL (p = 0.0075), free follicular 25(OH)D3 level > 0.00175 (p = 0.0146), and follicle size > 19.5 mm (p < 0.001) were all indicative of TQEs (Fig. 1).

## Discussion

### Principal findings

In this study, the total follicle 25(OH)D3 and follicular free 25(OH)D3 levels were higher in the TQE group compared with that in the non-TQE group. Our results were consistent with those of Alawad et al. Intrafollicular vitamin D levels were positively correlated with oocyte maturity, fertilization rate, and TQEs^14^. Arnanz et al. found that total follicle 25(OH)D3 levels and follicular free 25(OH)D3 levels were predictors of euploid embryos in the vitamin D-deficient group^13^; however, a study by Ciepiela et al. found different results, in which the TQE group had lower follicular 25(OH)D3 levels compared with the non-TQE group^4^. Rudick et al. found that race affected the relationship between vitamin D status and post-IVF pregnancy rates^15^. The results of this study were consistent with those of Zainab M. Alawad and Arnanz et al. because of the same sample types (Asian racial group), whereas in the study by Ciepiela et al., the subjects were Hispanic.

A study conducted by Cai et al. showed that total serum 25(OH)D3 levels correlated with serum-free 25(OH)D3 levels^16^. In this study, total follicle 25(OH)D3 levels correlated with follicular free 25(OH)D3 levels; however, in the present study, total follicular 25(OH)D3 levels were not correlated with total serum 25-(OH)-vitamin D3 levels. This is in contrast to the study by Liu et al., in which the levels of vitamin D in follicular fluid correlated with serum vitamin D levels^15^ These results may be related to the pleiotropic effect of vitamin D, which is associated with the vitamin D response element gene and yields a different phenotype in each cell.

The median follicular calcium levels in the TQE group were higher compared with those in the non-TQE group [6.30 (3.74–18.49) mg/dL vs 6.23 (3.60–17.82) mg/dL, respectively], but they were not significantly different (p = 0.805). Follicular calcium levels were positively associated with serum 25(OH)D3 levels in the TQE group (r = 0.218, p = 0.046) in all study samples (r = 0.216, p = 0.004); however, calcium levels did not correlate with serum 25(OH)D3 levels in the non-TQE group (r = 0.051, p = 0.216). Follicular calcium levels were positively correlated with serum calcium levels in the TQE group (r = 0.222, p = 0.040), the non-TQE (r = 0.215, p = 0.046), and all study samples (r = 0.224, p = 0.003). The results of this study are consistent with those of Ozdamar et al., in which follicular calcium levels in the IVF group in pregnant vs. nonpregnant patients were not significantly different^17^. It was hypothesized that during follicular fluid sampling, a specific calcium oscillation process did not occur, so there was no activation of intracellular calcium ions.

Patients with total follicular 25(OH)D3 levels >12.1 ng/mL had a 2.38-fold higher tendency to achieve TQE. Ciepiela et al. found that the cutoff value of serum 25(OH)D3 as a predictor of TQEs was > 12.7 ng/mL^4^. Liu et al. reported a serum 25(OH)D3 cutoff value >14.1 ng/mL, which predicts a normal fertilization rate, pregnancy rate, and live birth rate^15^. The cutoff value of follicular free 25(OH)D3 levels, which may be used as a predictor of TQEs, was >0.00175 ng/mL [95% CI 0.605 (0.528–0.678), p = 0.0146, sensitivity 34.88%, specificity 85.06%]. The relationship between the cutoff value of follicle-free 25(OH)D3 levels >0.00175 ng/mL between the TQE and non-TQE groups were significantly different [p = 0.002, OR (95% CI) 3.05(1.46–6), 38)]. Patients with follicle-free 25(OH)D3 levels >0.00175 ng/mL had a 3.05-fold higher tendency to produce TQE.

The mechanism that may explain the results of this study is that follicular free 25(OH)D3 affects AMH signaling and steroidogenesis in granulosa cells, which enables good follicle development to occur. Modulation of steroidogenesis in granulosa cells through the activation of the AMP-activated protein kinase (AMPK) pathway increases follicle maturation. Vitamin D also regulates oxidative stress at the cellular level, which plays an important role in follicular development. Mature oocytes can fertilize and form high-quality embryos.

The study had several limitations, including the lack of a correlation between serum and follicular fluid vitamin D levels. Also, we did not specifically analyze the significance of the relationship between serum AMH levels and follicular vitamin D levels. In addition, the study did not directly determine whether higher follicular vitamin D levels enhance the chances of achieving pregnancy.

## Conclusion

Free 25(OH)D3 and calcium levels in follicular fluid act independently during steroidogenesis, folliculogenesis, and fertilization. Further studies to determine a correlation between free 25(OH)D3 levels in follicular fluid and serum with euploid embryo status, clinical pregnancy rate, and live birth rate are needed. Additional sequelae studies are needed to determine whether vitamin D3 supplementation improves oocyte and embryo quality, as well as the correlation of calcium ion levels in granulosa cells with embryo quality.

## Data Availability

The dataset used and/or analysed during the current study available from the corresponding author on reasonable request.

## Abbreviations

25(OH)D3: 25-hidroxy-vitamin D3
AFC: Antral follicle count
AMH: Anti-mullerian hormone
IVF: In vitro fertilization
IGF-1: Insulin-like growth factor-1
IGFBP-1: Insulin-like growth factor-binding protein-1
VDBP: Vitamin D-binding protein
VDR: Vitamin D receptors

## References

[1] Vander Borght, M. & Wyns, C. Fertility and infertility: Definition and epidemiology. *Clin. Biochem.* **62**, 2–10 (2018). 29555319 10.1016/j.clinbiochem.2018.03.012

[2] Carson, S. A. & Kallen, A. N. Diagnosis and management of infertility: A review. *JAMA* **326**(1), 65–76 (2021). 34228062 10.1001/jama.2021.4788 PMC9302705

[3] Basile, S. et al. *Vitamin D and infertility: A narrative review.*

[4] Ciepiela, P. *et al.* Vitamin D as a follicular marker of human oocyte quality and a serum marker of in vitro fertilization outcome. *J. Assist. Reprod. Genet.* **35**, 1265–1276 (2018). 29774457 10.1007/s10815-018-1179-4 PMC6063829

[5] Muscogiuri, G. *et al.* Shedding new light on female fertility: The role of vitamin D. *Rev. Endocr. Metab. Disord.* **18**, 273–283 (2017). 28102491 10.1007/s11154-017-9407-2

[6] Skowrońska, P. *et al.* The role of vitamin D in reproductive dysfunction in women-a systematic review. *Ann. Agric. Environ. Med.* **23**(4), 671–676 (2016). 28030942 10.5604/12321966.1226865

[7] Hosseinisadat, R. *et al.* Assessment of the effect of serum and follicular fluid vitamin D and glucose on assisted reproductive technique outcome: A cross-sectional study. *Int. J. Reprod. BioMed.* **20**(3), 221 (2022). 35571501 10.18502/ijrm.v20i3.10714 PMC9099362

[8] Ko, J. K. *et al.* 100 years of vitamin D: Effect of serum vitamin D level before ovarian stimulation on the cumulative live birth rate of women undergoing in vitro fertilization: A retrospective analysis. *Endocr. Connect.*10.1530/EC-21-0444 (2022). 35029541 10.1530/EC-21-0444 PMC8859949

[9] Thomson, R. L., Spedding, S. & Buckley, J. D. Vitamin D in the aetiology and management of polycystic ovary syndrome. *Clin. Endocrinol.* **77**(3), 343–350 (2012). 10.1111/j.1365-2265.2012.04434.x 22574874

[10] Jensen, M. B. Vitamin D and male reproduction. *Nat. Rev. Endocrinol.* **10**(3), 175–186 (2014). 24419359 10.1038/nrendo.2013.262

[11] Cozzolino, M. *et al.* How vitamin D level influences in vitro fertilization outcomes: Results of a systematic review and meta-analysis. *Fertil. Steril.* **114**(5), 1014–1025 (2020). 33012554 10.1016/j.fertnstert.2020.05.040

[12] Grzeczka, A. *et al.* Relevance of vitamin D and its deficiency for the ovarian follicle and the oocyte: An update. *Nutrients* **14**(18), 3712 (2022). 36145088 10.3390/nu14183712 PMC9502977

[13] Chun, R. F. & Nielson, C. M. Free vitamin D: Concepts, assays, outcomes, and prospects. *Vitamin D*10.1016/B978-0-12-809965-0.00051-3 (2018).

[14] Alawad, Z. M. Level of follicular fluid vitamin D and embryo quality in a sample of Iraqi women undergoing IVF. *J. Fac. Med. Baghdad* **60**(4), 215–221 (2018).

[15] Arnanz, A. *et al.* Vitamin D in follicular fluid correlates with the euploid status of blastocysts in a vitamin D deficient population. *Front. Endocrinol.* **11**, 609524 (2021). 10.3389/fendo.2020.609524 PMC7874043 33584542

[16] Monastra, G. *et al.* Vitamin D: A steroid hormone with progesterone-like activity. *Eur. Rev. Med. Pharmacol. Sci.* **22**(8), 2502–2512 (2018). 29762856 10.26355/eurrev_201804_14845

[17] Rudick, B. *et al.* Characterizing the influence of vitamin D levels on IVF outcomes. *Hum. Reprod.* **27**(11), 3321–3327 (2012). 22914766 10.1093/humrep/des280

